# Quantitative Biomarkers for Cancer Detection Using Contrast-Free Ultrasound High-Definition Microvessel Imaging: Fractal Dimension, Murray’s Deviation, Bifurcation Angle & Spatial Vascularity Pattern

**DOI:** 10.1109/TMI.2021.3101669

**Published:** 2021-11-30

**Authors:** Redouane Ternifi, Yinong Wang, Eric C. Polley, Robert T. Fazzio, Mostafa Fatemi, Azra Alizad

**Affiliations:** Department of Physiology and Biomedical Engineering, Mayo Clinic College of Medicine and Science, Rochester, MN 55905 USA.; Department of Radiology, Mayo Clinic College of Medicine and Science, Rochester, MN 55905 USA; Department of Biomedical Engineering, Southern Medical University, Guangzhou, Guangdong 510515, China.; Department of Health Science, Mayo Clinic College of Medicine and Science, Rochester, MN 55905 USA.; Department of Radiology, Mayo Clinic College of Medicine and Science, Rochester, MN 55905 USA.; Department of Physiology and Biomedical Engineering, Mayo Clinic College of Medicine and Science, Rochester, MN 55905 USA.; Department of Radiology, Mayo Clinic College of Medicine and Science, Rochester, MN 55905 USA.

**Keywords:** Bifurcation angle, contrast-free ultrasound microvessel imaging, microvessel fractal dimension, Murray’s deviation, spatial vascular pattern

## Abstract

A growing body of evidence indicates that there is a strong correlation between microvascular morphological features and malignant tumors. Therefore, quantification of these features might allow more accurate differentiation of benign and malignant tumors. The main objective of this research project is to improve the quantification of microvascular networks depicted in contrast-free ultrasound microvessel images. To achieve this goal, a new series of quantitative microvessel morphological parameters are introduced for differentiation of breast masses using contrast-free ultrasound-based high-definition microvessel imaging (HDMI). Using HDMI, we quantified and analyzed four new parameters: 1) microvessel fractal dimension (mvFD), a marker of tumor microvascularcomplexity; 2) Murray’sdeviation(MD), the diameter mismatch, defined as the deviation from Murray’s law; 3) bifurcation angle (BA), abnormally decreased angle; and 4) spatial vascular pattern (SVP), indicating tumor vascular distribution pattern, either intratumoral or peritumoral. The new biomarkers have been tested on 60 patients with breast masses. Validation of the feature’s extraction algorithm was performed using a synthetic data set. All the proposed parameters had the power to discriminate the breast lesion malignancy (p < 0.05), displaying BA as the most sensitive test, with a sensitivity of 90.6%, and mvFD as the most specifictest, with a specificityof 92%. The results of all four new biomarkers showed an AUC = 0.889, sensitivity of 80% and specificity of 91.4% In conclusion, the added value of the proposed quantitative morphological parameters, as new biomarkers of angiogenesis within breast masses, paves the way for more accurate breast cancer detection with higher specificity.

## I. Introduction

NEOVASCULARIZATION plays an essential role in cancer growth and metastasis [1], [2]. Low oxygen levels in early emerging tumors produce a high quantity of angiogenetic growth factors that initiate angiogenesis [3], [4]. The newly grown microvessels in malignant tumors are randomly and heterogeneously shaped, presenting with high density, leakiness, irregularity, and tortuous and larger structures [5]-[8]. Quantitative information of morphological parameters of microvasculature has been studied using imaging tools such as CT and MRI [8]-[10]. Analysis of tumor microvessel morphology, such as tortuosity, vessel diameter, vessel density, number of branching points and vessel segments, as biomarkers of malignancy, is challenging in ultrasound Doppler imaging, and has been investigated in contrast free Doppler imaging [11]. Using contrast agents, quantification of microvessel tortuosity has been investigated in preclinical studies by acoustic angiography method [12], [13]. Recent advances in Doppler imaging, using high frame rate ultrasound and new clutter removal processing methods, have made it possible to visualize small vessel structures [14]-[16]. Previously, our group developed a high-definition ultrasound microvessel imaging technique, resulting in enhanced vessel images from tumors at a high frame rate without injection of a contrast-enhancing agent [16]. Our group has also introduced tools, that prepare the images for quantification of vessel structures using a set of processing steps for morphological filtering and segmentation [11]. Our initial results show that quantitative assessment of microvessel morphological features extracted from high definition ultrasound imaging allows for differentiation of malignant from benign breast lesions (p-value < 0.005) and thyroid nodules (p-value < 0.01) [11].

While a multitude of metrics for analyzing microvascular architecture in contrast-free ultrasound imaging have been introduced previously [11], further development of new parameters is of paramount importance for improved differentiation of malignant from benign tumors. The four new biomarkers are described in the following paragraphs.

### A. Spatial Vascularity Pattern

Though increased vessel density of tumors is a reliable marker of tumor angiogenesis [17], classifying the pattern of microvessel distribution, either concentrated centrally inside or around the tumor periphery, is of paramount importance in differentiating malignant from benign tumors. For this purpose, the spatial vascularity pattern (SVP) parameter has been introduced as an important factor to label microvessel distribution, either as peripheral or intratumoral, in contrast-enhanced ultrasound Power Doppler imaging, for differentiation of benign from malignant thyroid nodules [18]. The SVP parameter provides global information about tumor microvessels as well as localization of blood vessels within the lesions, which can be used to separate benign from malignant. SVP assesses the tumor vascular distribution pattern as being either intratumoral, which is more concentrated inside, or being peritumoral, which is more concentrated peripherally. In the present work, we propose a new methodology to improve the SVP calculation for microvessel distribution patterns in contrast-free ultrasound microvessel imaging for differentiation of breast masses.

### B. Microvessel Fractal Dimension

It has been shown that the degree of complexity and irregularity of tumor microvessel could be used as a reliable factor in reflecting tumor angiogenesis [19]. Microvessel Fractal dimension (mvFD), a marker of tumor microvascular complexity, was first proposed as a potential biomarker of branching complexity for human retinal vessel analysis in patients with neurodegenerative disease and stroke using optical coherence tomography imaging technique [20]-[23]. In this paper, mvFD is first introduced as a biomarker in a contrast-free high definition ultrasound microvessel imaging for differentiation of benign and malignant breast masses.

### C. Bifurcation Angle

Abnormal bifurcation angle (BA) is found to be a valuable biomarker for the diagnosis of retinopathy and other chronic diseases using optical coherence tomography [24]. In this paper, BA is first introduced as a new biomarker in a contrast-free high definition ultrasound microvessel imaging for differentiation of benign and malignant breast masses.

### D. Murray’s Deviation

Specific quantification metrics, such as microvessel diameter, may carry unique significance, depending on the location analyzed within the tumor microvessel. For instance, altered pericyte function due to low oxygenation level changes microvessel diameter, locally, in a vessel segment [25]. In this work, we propose a new parameter, Murray’s deviation (MD), based on Murray’s law (ML) [26], for the estimation of diameter per vessel segment. Murray’s law describes the blood vessel geometry at a bifurcation according to the relation $(D_{daughter1}^{3}+D_{daughter2}^{3})∕D_{mother}^{3}=1$, where *D*_*daughter*1_ and *D*_*daughter*2_ represent the diameters of the two daughter vessels and Dmother is the diameters of the mother vessel [27]. ML has been described for the effect on branching geometry in large vessels [28], [29] and capillaries [30]. The diameter mismatch is defined as the deviation from Murray’s law. Diameter mismatch is found to be elevated in the tumor network relative to normal networks [31]. Also, the vascular network of retina in patients with diabetes mellitus may show a deviation from ML, when the daughter vessels are wider than predicted by ML, as reported in [32]. To the best of our knowledge, this is the first report of the use of MD for differentiation and classification of malignant and benign breast masses, using contrast-free high-definition ultrasound microvasculature imaging.

The focus of this paper is to introduce novel quantitative biomarkers for morphometric analysis of microvasculature networks using contrast-free ultrasound imaging. We have developed image processing techniques to automatically measure the microvessel fractal dimension, bifurcation angle, Murray’s deviation, and spatial vascularity pattern of the tumor microvessel network reconstructed from two-dimensional (2D) ultrasound in-phase and quadrature (IQ) data. Rigorous and complete validation of the feature’s extraction algorithm has been performed, using a synthetic data set. The paper also investigates the potential utility of these parameters in the differentiation of breast masses.

The rest of the paper is organized as follows: In Section II we discuss our methods and related work behind the proposed methods, as well as new quantification parameters, and explain our proposed algorithms in detail. In Section III we show our results from validation of our algorithms by simulation and in vivo studies. We end with a discussion and conclusion in Section IV.

## II. Materials and Methods

In the present work, we introduced four new parameters: microvessel fractal dimension, Murray’s deviation, bifurcation angle and spatial vascularity pattern to quantify the morphological features of contrast-free ultrasound microvasculature images for breast tumor classification. Simulation and in vivo studies were conducted to validate our algorithms.

### A. New Vessel Quantification Parameters

#### 1) Microvessel Fractal Dimension (mvFD):

**Fig. 1.**
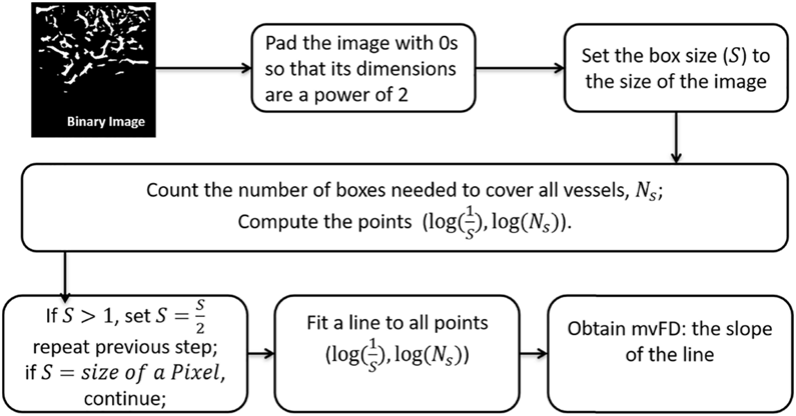
Diagram of microvessel fractal dimension calculation.

Microvessel fractal dimension is a metric used to quantify the structural complexity of a vascular network. The box counting method [33] was employed to calculate mvFD using the binary image through the following six steps as shown in the diagram in Fig. 1: 1) the binary image was padded with zeros to make its dimensions to a power of 2; 2) the box size (*S*) was set to the size of the binary image; 3) the number of boxes (*N_S_*) needed to cover all vessels (non-zero pixels) in the binary image were counted; 4) *S* was set to *S*/2 and step 3 was repeated only if *S* > size of a pixel, otherwise, the process continues to step 5; 5) a first-order polynomial fit was applied to the pairs, ($\log\left(\frac{1}{S}\right)$, log(*N_S_*)); 6) the slope of the fitted line was obtained as mvFD:

(1) $$mv\;FD=\underset{S\to O}{\text{lim}}\frac{\log\;N_{S}}{\log\;1∕s}$$

#### 2) Murray’s Deviation (MD):

To quantify vessel morphological parameters, binary and skeleton images were formed [11]. Finding the skeleton is based on a thinning algorithm [11], [34]. In this approach, vessels are sequentially thinned, and the midline of each vessel determined to construct the skeleton of a vessel network. Using skeleton image, we removed the 3-by-3 neighborhood pixels for each branch point to isolate and label each vessel component. This allowed us to determine the number of branches [11].

**Fig. 2.**
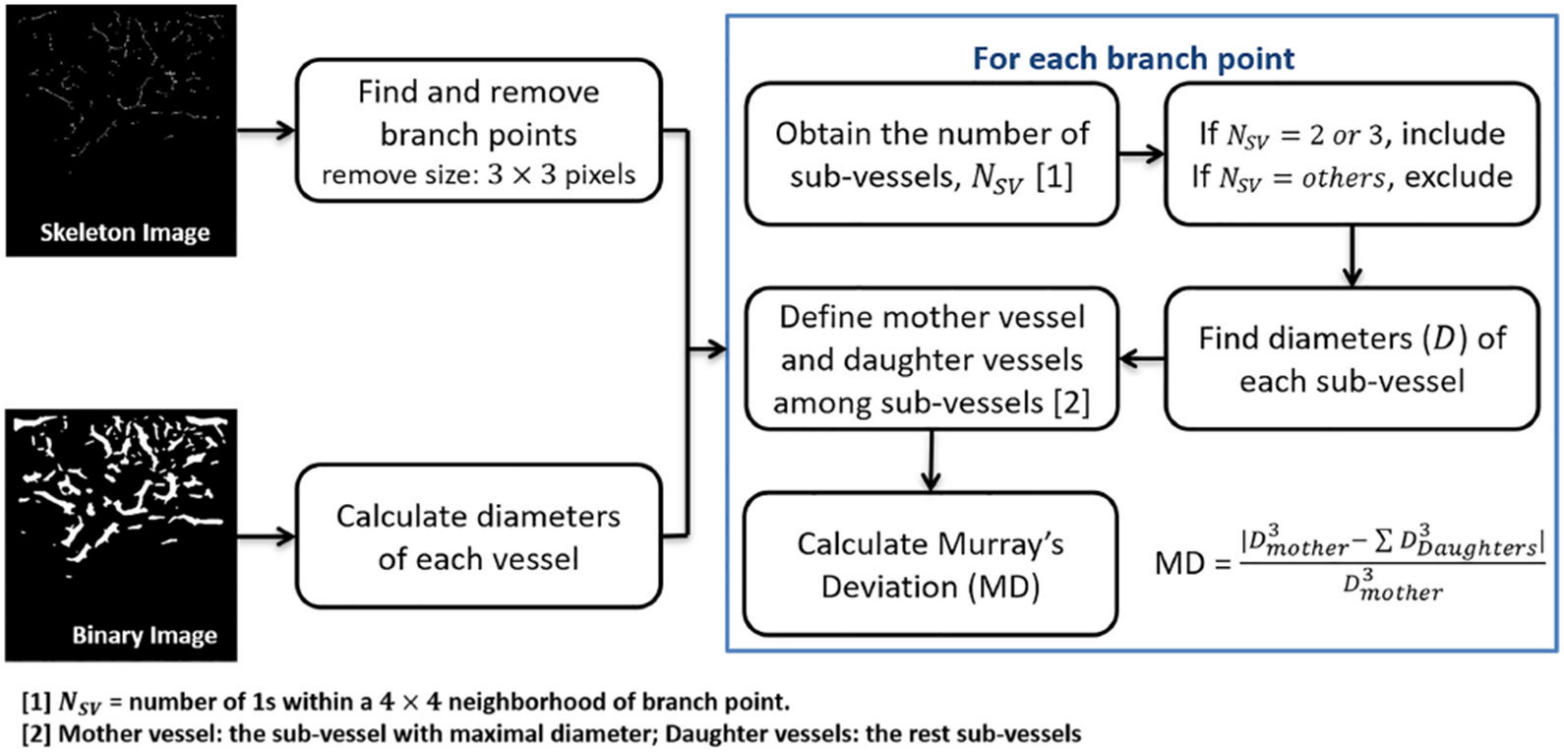
Diagram of Murray’s deviation calculation.

A unique label was assigned to each segment. Diameters (*D*) of vessels were calculated using the binary image. For each branch point, a 4 × 4-pixel neighboring region was defined on the skeleton image. The number of non-zero pixels was obtained as the number of sub-vessels (*N_SV_*). According to their labels, the diameters of sub-vessels were found from diameter data and then used to define mother and daughter vessels; the sub-vessel with the largest diameter was designated as a mother vessel and the remaining sub-vessels served as daughter vessel(s). MD was calculated using (2). A flowchart of the MD calculation is shown in Fig. 2.

(2) $$MD=\frac{|D_{mother}^{3}-\sum D_{daughter}^{3}|}{D_{mother}^{3}}$$

#### 3) Bifurcation Angle (BA):

**Fig. 3.**
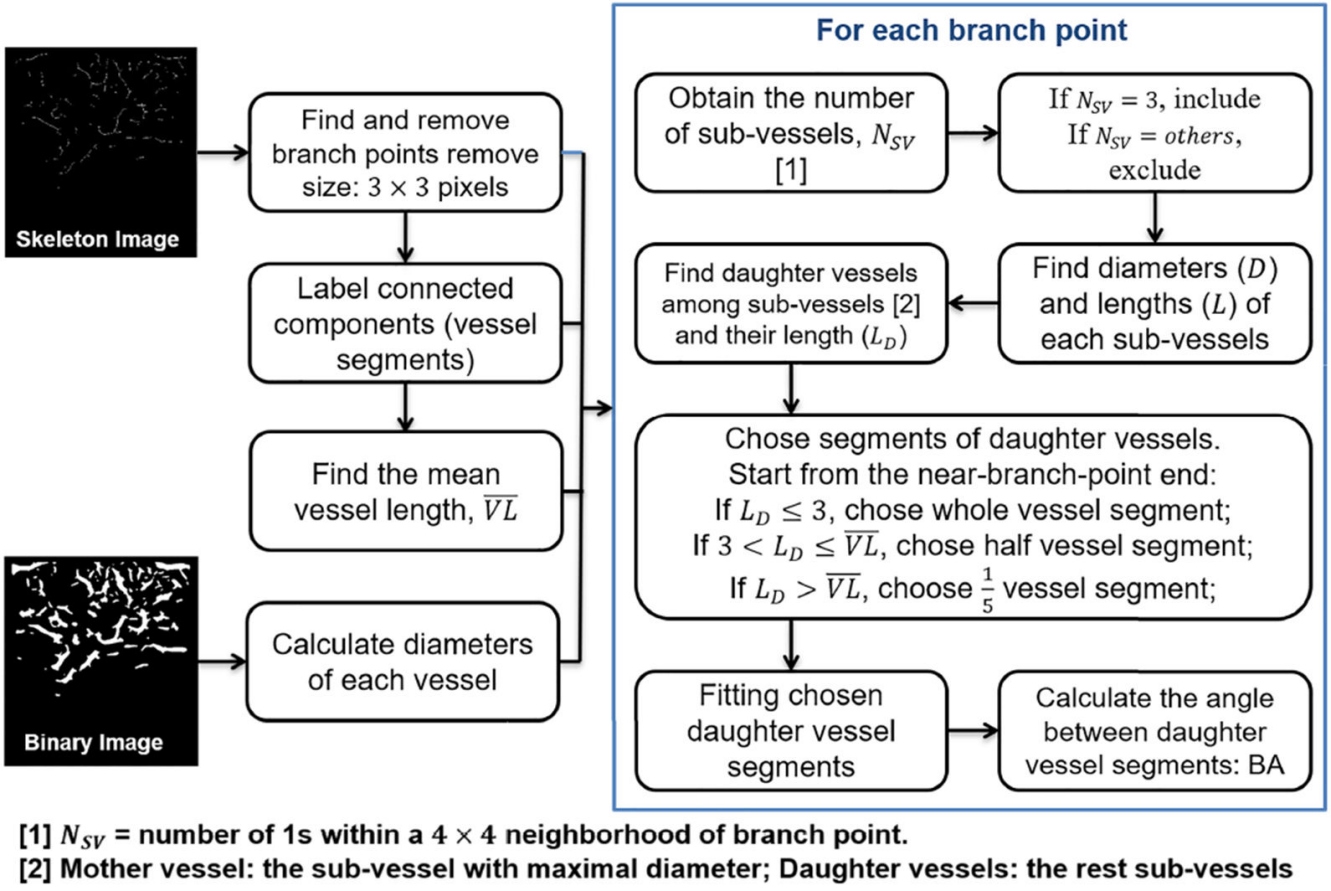
Diagram of bifurcation angle calculation.

Similar to the MD calculation, *N_SV_* was defined for each branch, but only the branch with three sub-vessels, one mother vessel and two daughter vessels, was included, as the BA refers to the angle between two daughter vessels. The average vessel length ($\bar{VL}$, unit pixels) of each data set was calculated after branch point removal. The two daughter vessels were selected the same way as was done for MD. Two straight lines were generated by fitting two daughter vessels, and the angle between them was calculated as BA. The irregularity of breast cancer blood vessels can introduce errors to the fitting operation. Thus, only the nearbranch-point part of the daughter vessels was used for fitting, based on their length (*L_D_*, unit pixels): if *L_D_* ≤ 3 pixels, whole daughter vessels were used; if $3<L_{D}\leq \bar{VL}$ pixels, half daughter vessels were used; if $L_{D}\;>\;\bar{VL}$ pixels, 1/5 daughter vessels were used (Fig. 3).

#### 4) Spatial Vascularity Pattern (SVP):

The spatial vascularity pattern assesses the tumor vascular distribution pattern as being either intratumoral, which is more concentrated inside, or being peritumoral, which is more concentrated peripherally, shown with two possible values (SVP = 1) and (SVP = 0), respectively. In the previous study [18], the SVP value was determined by first calculating the intensity profiles of the entire image and then normalized. After interpolating the results by a second order polynomial, the first derivative was calculated. If the first derivative was either always positive, or always negative, or simply monotonically increasing, the SVP value was considered equal to zero (peritumoral vascularity), otherwise the SVP value is equal to 1 (intra-tumoral vascularity).

**Fig. 4.**
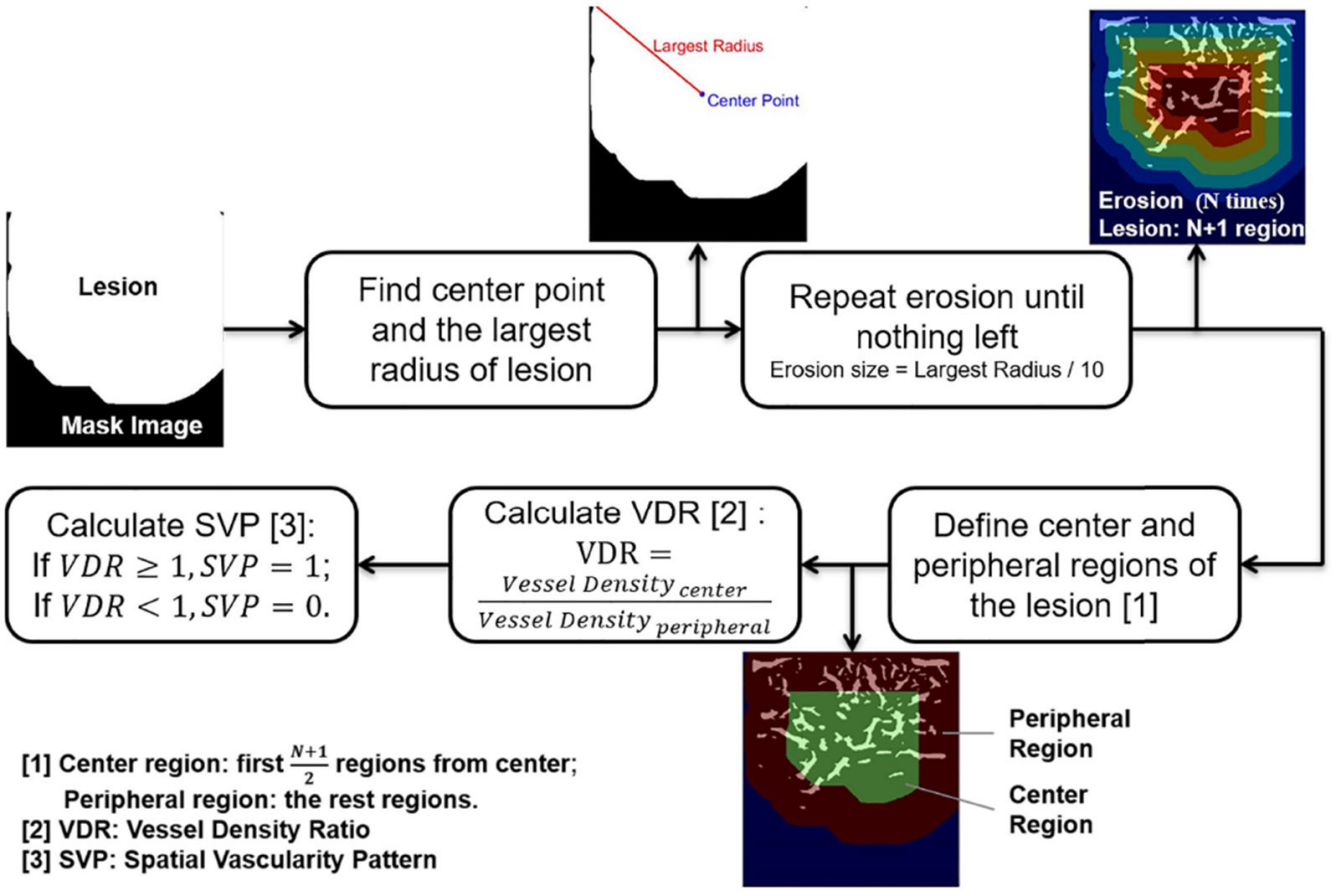
Diagram of spatial vascularity pattern calculation.

In the present study, to calculate the SVP, we performed image erosion to mask the image, then the geometric center of the lesion was defined, and the largest radius was selected on the mask image. Next, after defining the center and peripheral regions of the lesion, the vessel density ratio (VDR) was calculated. A flowchart of the SVP calculation is shown in Fig. 4.

The vessel density ratio (VDR) has been previously defined [35]. In this study, we have defined VDR as a ratio of vessel density of the tumor center to periphery. Therefore, VDR can describe the tumor vessel distributions at the periphery (VDR < 1), or at the center (VDR > 1) or both (VDR ≈ 1). VDR was calculated as:

(3) $$VDR=\frac{Vessel\;Density_{center}}{Vessel\;Density_{peripheral}}$$

Finally, after calculating VDR, we have defined SVP as:

(4) {VDR≥1,SVP=1VDR<1,SVP=0}

A VDR equal to or larger than 1 defines the SVP value as equal to 1, meaning the vascular distributions are more concentrated centrally and a VDR less than 1 defines the SVP value as zero, meaning the vascular distributions are more concentrated peripherally.

### B. Simulation Study

To validate the algorithms that are developed for the quantification of morphological parameters, we conducted the following simulation study. To validate the algorithms that are developed for the quantification of morphological parameters, we conducted the following study. First, using AutoCAD 2017 software (Autodesk, San Rafael, CA), we create multiple simulated models of microvessel network with different complexities and different sets of morphological parameters.

**Fig. 5.**
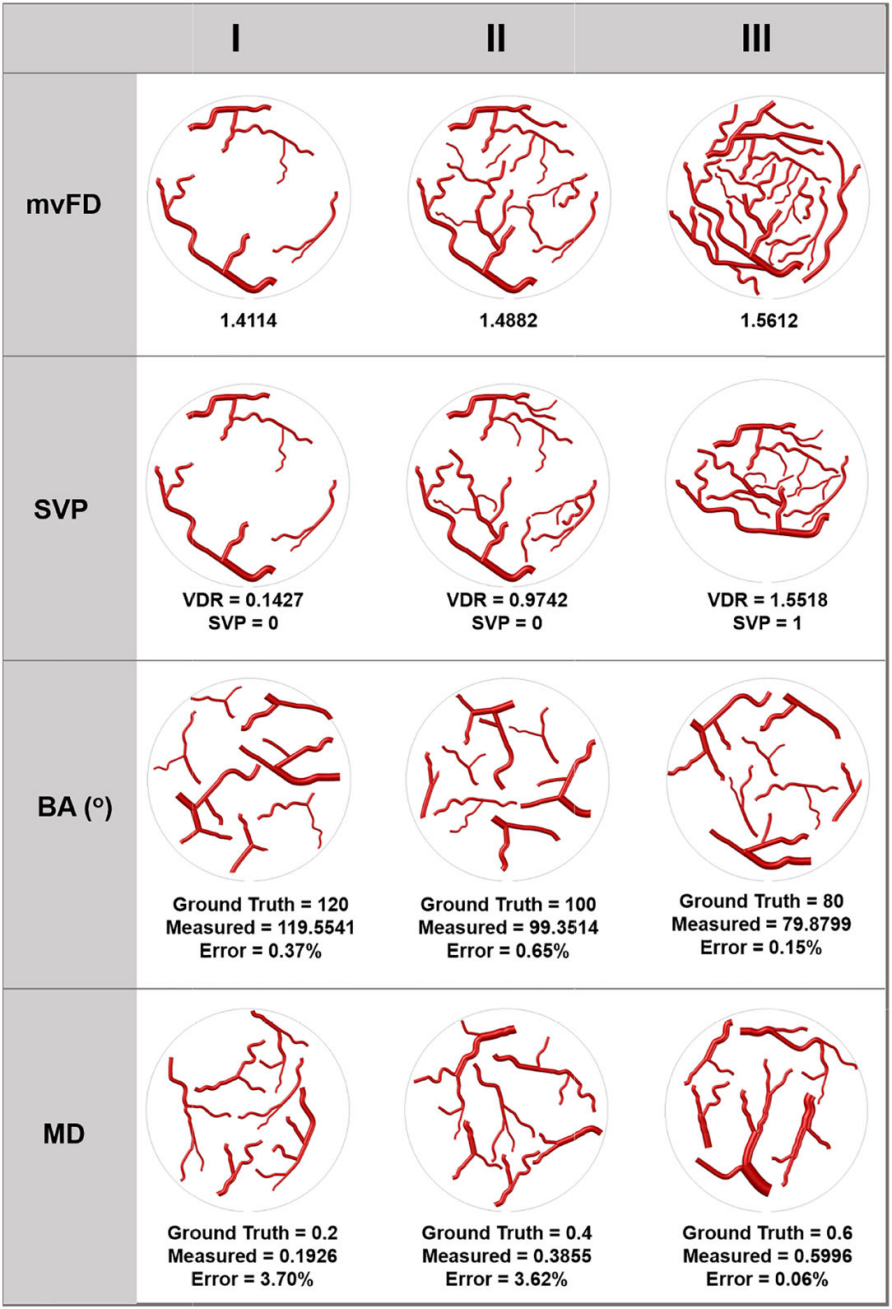
An illustration of twelve simulated small blood vessel networks for the analysis of the four new morphological parameters. **First row** represents different microvessels complexity in terms of fractal dimension (mvFD) with increasing complexity from column I, to Column III. **Second row** represents the distribution of microvessels based on spatial vascular pattern (SVP), as shown peripherally concentrated in column I and II, and centrally concentrated in Column III. **Third row** represents variations of bifurcation angles **(BA)** that decreases from Column I to II and III and the measurements are compared to the ground truth., **Fourth row** shows variations of Murray’s deviation (MD), where the diameter mismatch increases from columns I to II and III. The measured MD values are compared to the ground truth.

Then, using the simulated microvessel models, we test the accuracy of the morphological parameter estimation algorithm by comparing the estimated parameters against the known values (ground truth) of the simulated models. The first row in Fig. 5, presents simulated vessel structures, with increasing vascular complexity from columns I to II and III, for mvFD estimation. In the second row of Fig. 5, peripherally concentrated simulated vessels (column I and II) and centrally concentrated vessels (column III) are shown. Based on our patient results, mean BA values of simulated vessels in Fig. 5, third row, columns I, II and III, were preset to 120°, 100° and 80°, respectively. For this purpose, we designed each simulated model with different angles assigned to different bifurcations such that the mean BA of the model equaled the desired preset value. Similarly, mean MD values of simulated vessel models in Fig. 5, fourth row, columns I, II and III were preset to 0.2, 0.4 and 0.6, respectively. The preset values for BA and MD were used as ground truth. To validate our method, the measured BA and MD values were compared to the ground truth.

All vessel morphological features and distributions, displayed in Fig. 5, were used to validate the developed algorithms for in vivo estimation of the proposed microvessel morphological parameters.

### C. Patient Study

Under the Mayo Clinic Institutional Review Board (IRB) approved protocol, a total of 60 patients, with age range 18 to 87 years (mean age 51 ± 16 years) with ultrasound-identifiable breast lesions were recruited for our study. IRB-approved signed written consent was obtained from each patient. All patients underwent core needle breast biopsy following ultrasound examination, and pathology results were used as the final diagnosis. From a total of 60 breast tumors, the pathology revealed 35 were benign and 25 malignant. Furthermore, the lesion size in the largest dimension ranged from 5 mm to 40 mm with a mean of 15.86 ± 7.81 mm.

### D. Image Acquisition and Processing

In vivo imaging of breast lesion microvessels was obtained using the high-definition microvessel imaging (HDMI) contrast-free ultrasound technique developed by our group [16]. An Alpinion Ecube12-R ultrasound machine (ALPINION Medical Systems, Seoul, South Korea), with a linear array operating at 8.5MHz, L3-12H (ALPINION Medical Systems) was used for image acquisition of breast tumors. B-mode images of breast tumors were identified using the plane-wave imaging mode, then a sequence of high frame rate data (at ~600 frames per second) was acquired on the breast tumor site. All processing was performed offline using MATLAB RRID:SCR_001622 (Mathworks, Natick, MA). Image formation of the HDMI technique was first completed using clutter filtering, background noise reduction and a series of filtering image enhancement techniques that automatically extract vessel architecture [16]. Subsequently, morphological filtering, vessel segmentation, and skeletonization were applied to obtain the final clean binary images [11]. Images of the microvasculature networks were further analyzed to extract new quantitative parameters. The study team that performed the image and data analysis was blind to the breast biopsy and clinical data. The method for calculation of each parameter is presented in the vessel quantification section.

### E. Vessel Quantification

Breast lesions were first segmented using B-mode images reconstructed from the IQ data. To quantify vessel morphological parameters, binary and skeleton images were formed [11]. The new parameters, including MD, BA, VDR, SVP and mvFD, were extracted from the output images and then analyzed.

### F. Statistical Analysis Methods

For each parameter, diagnostic performance for differentiation of benign and malignant breast lesions was determined using receiver operating characteristics (ROC) analysis. The Wilcoxon rank-sum test using R software (version 3.6.2) was applied to investigate specificity, sensitivity and area under the curve (AUC), considering 95% confidence for all parameters. Statistical diagnostic potential for discriminating breast lesions with a p-value < 0.05 was considered significant. In addition, Pearson’s Chi-squared test was used for binary data.

## III. Results

### A. Simulation Results

The estimates of mvFD in simulated vessel structures were 1.4114, 1.4882 and 1.5612, in columns I to II and III of Fig. 5, row 1, respectively, indicating a higher network complexity with wavier vessel shape and increased vessel segments in the simulated vessels. SVP values based on VDR calculation describe the vascular distribution patterns in the simulated vessels in the second row of Fig. 5. The estimated VDR for the simulated vessel patterns in column I and II, are 0.1427 and 0.9742, respectively, indicating SVP = 0 for the peritumoral distribution. Estimated VDR for the simulated vessel patterns in column III is 1.5518, indicating SVP = 1 for the intratumoral distribution. In the third row of Fig. 5, the measurements of BA parameters were 119.5541, 99.3514 and 79.8799 with errors of 0.37%, 0.65% and 0.15% in columns I, II and III, respectively. These BA measurements were almost equal to the ground truth of 120, 100 and 80, with the mean error of 0.39%. In Fig. 5, row 4, MD of simulated vessels were 0.1926, 0.3855 and 0.5996 with errors of 3.7%, 3.6% and 0.067% in columns I, I and III, respectively. These MD measurements were very close to the ground truth of 0.2, 0.4 and 0.6, with a mean error of 2.46%.

### B. In Vivo Study Results

#### 1) Histopathological Results:

In a total of 60 patients examined by HDMI, the pathology results of ultrasound guided core needle breast biopsy revealed 35 benign and 25 malignant breast lesions. In the total of 60 lesions, there were 43 lesions with the diameter ≤ 20 mm, and 17 lesions with the diameter > 20 mm. The histological types of benign lesions obtained from breast biopsy include, atypical (n = 1), ductal ectasia (n = 2), pseudoangiomatous stromal hyperplasia (PASH) (n = 3), fat necrosis (n = 3), papilloma (n = 4), benign changes (n = 5) and fibroadenoma (FA)(n = 17). The histologic types for malignant lesions include ductal carcinoma insitu (DCIS) (n = 2), and invasive ductal carcinoma (IDC) (n = 21).

#### 2) Quantification of Morphological Parameters of Breast Tumor Microvessel:

**Fig. 6.**
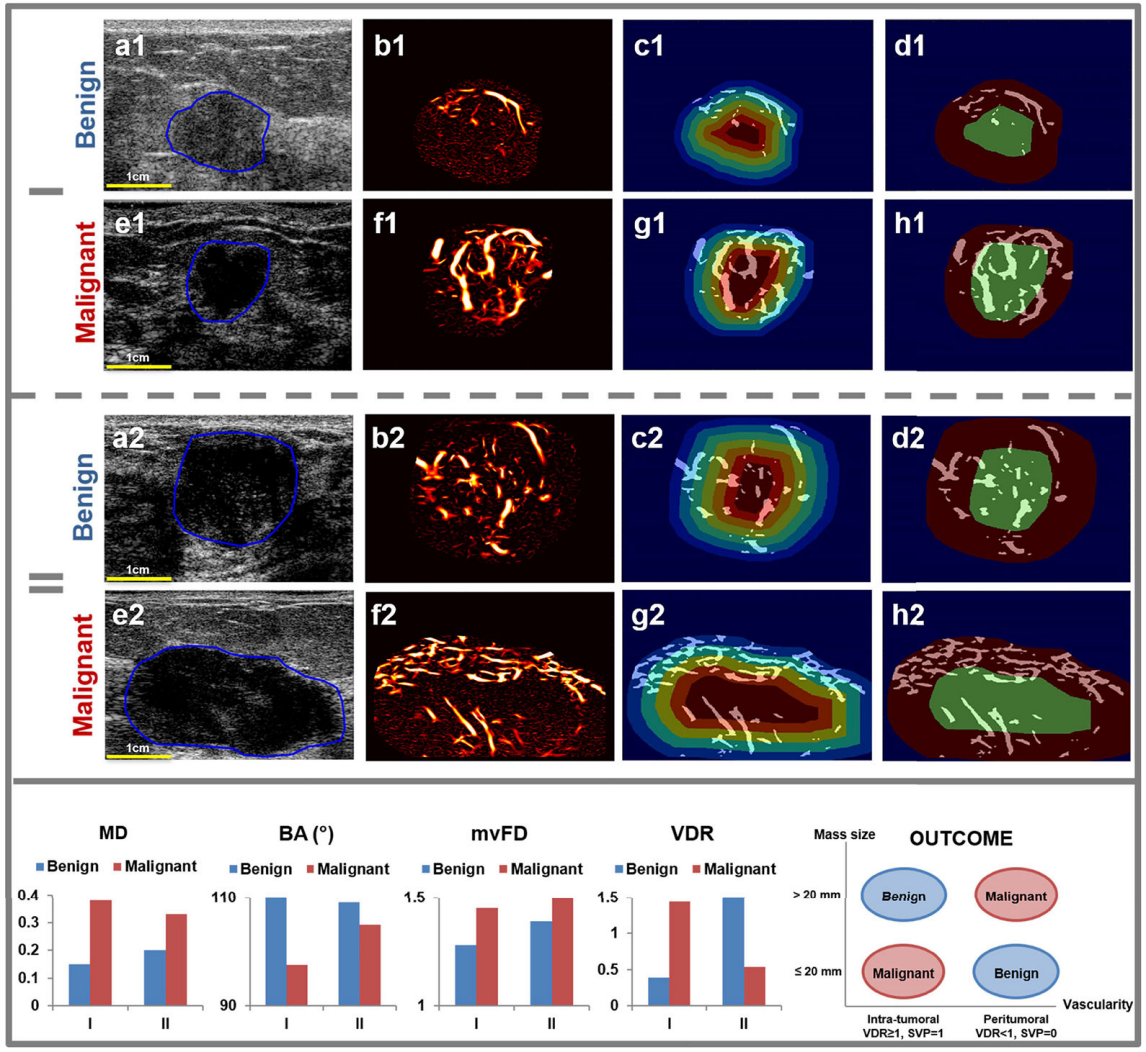
Quantitative results of breast lesion microvessels in four representative cases, two benign and two malignant based two size groups. Group I and II represent benign and malignant cases for lesion ≤ 20 mm and > 20 mm, respectively. B-mode images (a1, e2, a2 and e2), HDMI images (b1, f1, b2 and f2), SVP erosion (c1, g1, c2 and g2) and calculation (d1, h1, d2 and h2; green = center region, red = peripheral region) images were displayed. Bar graphs on the bottom row are the results of the new parameters for the two benign and two malignant breast masses. The diagram on right side of the bottom row shows the outcome of SVP based on breast tumors’ mass sizes, ≤ 20mm. and > *20mm*.

In vivo results of microvessel morphology of two benign and two malignant breast masses are shown in Fig. 6. Group I and II represent B-mode, HDMI with SVP diagrams for lesion diameter ≤ 20 mm and > 20 mm. Compared with the benign lesion, MD and mvFD for malignant lesions in each group are increased, indicating a deviation from optimized vascular branch structure and a higher vasculature complexity in the malignant lesions, respectively. A BA decrease in malignant lesions demonstrated a denser blood vessel distribution. The SVP factorial values, *SV P* = 0, indicating a peritumoral vascularity and *SV P* = 1, indicating an intratumoral vascularity pattern are used for differentiation of benign and malignant lesions. However, the lesion discrimination outcome is based on breast mass size. In breast masses 20mm or smaller, peripherally concentrated vascularity (VDR < 1) or SVP 0, is associated with a benign diagnosis, but in breast masses larger than 20mm it is associated with malignant diagnosis. In breast masses larger than 20mm, centrally concentrated vascularity (VDR > 1), or SVP 1, is associated with benign diagnosis, however in breast masses 20mm or smaller is associated with malignant diagnosis. The diagram on the right side of the bottom row of Fig. 6 illustrates the outcome of SVP based on the mass size group.

**Fig. 7.**
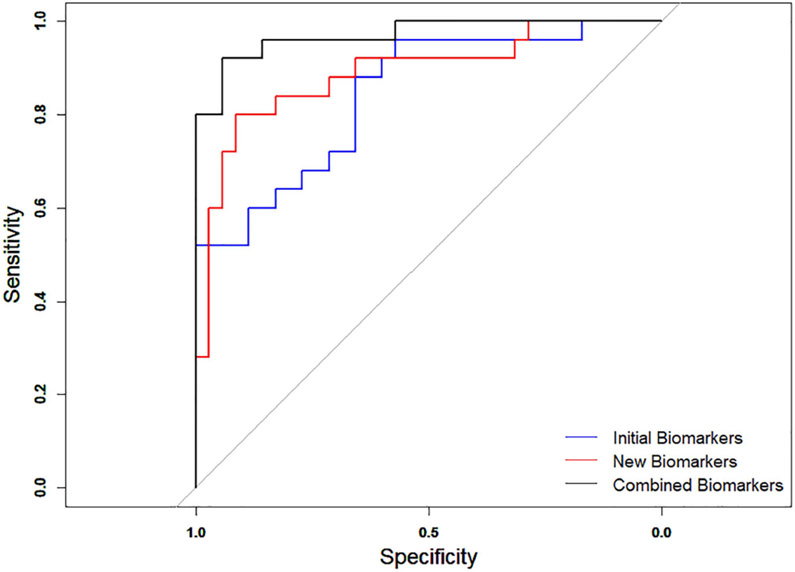
ROC curves generated for a total of 60 patients using the initial biomarkers (blue), new biomarkers (red) and combination of the initial and new biomarkers (black).

In the total of 60 patients, including 35 with benign and 25 with malignant breast lesions, the performance of the four new quantitative morphological parameters were tested and compared with that of our initial biomarkers [11]. The multivariable logistic regression analysis of our initial biomarkers (vessel density, tortuosity, diameter, vessel length, number of vessel segments and number of branches) showed an AUC = 84.11%, with Sen = 88% and Spec = 65.71%. The analysis of the newly proposed quantitative parameters (mvFD, SVP, BA, MD) showed an AUC = 88.91%, with Sen= 80% and Spec = 91.43%. The performance of the combined parameters (initial and new) showed an AUC = 97.03%, with Sen = 92% and Spec = 94.29%. the ROC curves are shown in Fig. 7.

**Fig. 8.**
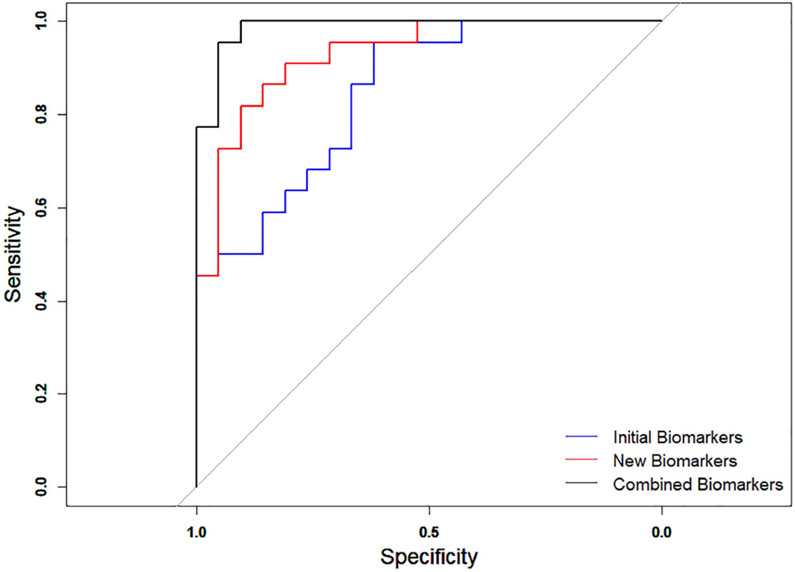
ROC curves generated for benign fibroadenoma and IDC using the initial biomarkers (blue), new biomarkers (red) and combined of the initial and new biomarkers (black).

The performance of the four new quantitative biomarkers were also tested on the HDMI images of two major histological types with highest sample size, IDC (malignant) and FA (benign), and compared with that of initial biomarkers. The numbers of patients within other histological types were too small for a meaningful statistical analysis. The multivariable logistic regression analysis of the initial biomarkers showed an AUC = 0. 846 and 95% CI:73.3-95.9 with sensitivity of 65.9% and specificity of 89.4. Analysis of the newly proposed quantitative parameters (mvFD, SVP, BA, MD) showed an AUC = 0.929 and 95% CI:85.4-100, with sensitivity of 81.8% and specificity of 90.5%. The performance of the combined parameters (initial and new) showed an AUC = 0.987, with sensitivity of 95.5% and specificity of 95.2%. The ROC curves are shown in Fig. 8.

Data are presented as mean ± standard deviation in Table I for the benign and malignant groups. All the proposed parameters had the power to discriminate the lesion malignancy (p < 0.05). Although, all the parameters performed well with AUC above 80%, the mean of MD was the best-performing with the AUC of 87.06%. Among all parameters, the MD was the most sensitive test with a sensitivity of 96%, and mvFD was the most specific test with a specificity of 92%. Although minimum MD and median and max BA were not statistically significant, the mean, max and median MD as well as the mean and minimum BA, showed statistical significance.

## IV. Discussion

This paper presents a new series of microvessel morphological parameters as quantitative tumor biomarkers for differentiation of malignant and benign breast lesions. Recently, our group developed a new contrast-free US-based high-definition microvessel imaging technique that reveals tumor microvessels in submillimeter size [16], and we complemented this technique with novel quantification tools to assess vessel morphological features, including vessel density, tortuosity, diameter, number of vessel segments and branch points [11]. To the best of our knowledge, mvFD, MD and BA are first introduced in this paper as new quantitative morphological parameters of tumor microvessels in a contrast-free ultrasound microvessel imaging for differentiation of breast masses. Moreover, a new method of SVP estimation is also presented for breast tumor differentiation. Synthetic data sets and in vivo images of breast tumor microvessels demonstrate the robustness and accuracy of these biomarkers.

The present study shows that Murray’s deviation was significantly higher in malignant than in benign breast lesions. To maintain the optimum blood flow in a branching vascular network, Murray’s law controls the vessel diameter [26]. On the other hand, the vascular network of diseased tissue could show a deviation from ML, Murray’s deviation, as investigated in this study. In previous studies, the diagnostic value of MD was demonstrated for different diseases, including coronary artery disease, calcifications and portal hypertension in patients with liver cirrhosis [36]-[39], Our study also demonstrates a higher MD in malignant breast lesions. To the best of our knowledge, our study is the first to classify breast masses using MD through contrast free ultrasound microvasculature imaging.

A previous study showed a strong inverse correlation between coronary artery BA and atherosclerotic lesion localization distance to the bifurcation site, suggesting BA as an independent risk factor for lesion localization [40]. On the other hand, our study found that BA is significantly decreased in malignant breast lesions. Similarly, another study showed a decreased BA in invasive carcinomas of the colon compared to that of in normal tissue [41].

The results of SVP in this study showed that a peripherally and centrally distributed vasculature is associated with malignancy in large breast lesions (diameter > 20 mm) and small breast lesions (diameter ≤ 20 mm), respectively. Similarly, studies reported that while small and early stage malignant tumors have rich centrally-distributed vascularity, as the tumors enlarge, as a consequence of rapid growth of malignant parenchyma, areas in the center may become necrotic, thus larger malignant tumors can be associated with low vascularity in central region but with peripherally-distributed microvasculature [35], [42]-[44]. Furthermore, studies on evaluation of breast tumor angiogenesis using contrast enhanced ultrasound (CEUS) showed that a peripherally distributed vasculature is associated with malignancy in large breast lesions, whereas malignancy results in a centrally distributed vasculature in case of small breast lesions [35], [42]. However, in these studies, the peripheral enhancement of vascular patterns was subjectively defined by physicians, based on CEUS images. In our study, using quantitative metrics of SVP in ultrasound-based contrast-free high-definition microvessel imaging, we confirmed that peripheral distribution of breast tumor microvessels is in favor of malignancy.

Fractal dimension measures the complexity of structures. In our study, a significant increase of mvFD was found in malignant breast lesions, indicating that malignant lesions have more dense, irregularly branched and twisted microvessel structures, which is consistent with the findings of other studies on oral cancer [45], renal cell carcinoma [19] and glioblastoma [46].

One limitation of our study is that all quantitative biomarkers were evaluated using 2D HDMI, The 2D method overlooks some important 3-dimensional (3D) morphological features and the connectivity of blood microvessels leading to either underestimation or overestimation of different morphological parameters including the new proposed parameters, MD, BA, mvFD and SVP, in a 2D plane. For example, the angle estimation in 2D is often underestimated compared to its true value in 3D. Volumetric assessment of microvessels’ morphological features require development of 3D HDMI, including 3D ultrasound data acquisition, 3D processing codes to extract the 3D morphological biomarkers, and 3D quantification algorithms. Another limitation of this study is the sample size that was relatively small. Thus, a 3D HDMI study on a larger patient population is required to further investigate the role of these new quantitative microvessel parameters in the differentiation of breast masses.

## V. Conclusion

In summary, in this paper, new quantitative microvessel morphological parameters were first introduced for differentiation of breast masses using contrast-free ultrasound-based high-definition microvessel imaging. Using HDMI, we quantified and analyzed four new parameters: microvessel fractal dimension, Murray’s deviation, branch angle and spatial vascular pattern on 60 patients with breast masses, where 25 were malignant and 35 benign. The proposed mvFD shows significance in differentiating malignant from benign breast tumor. We also observed that MD, BA, and SVP showed significant value in breast tumor differentiation. Future studies using contrast-free ultrasound-based quantitative HDMI with larger patient populations are warranted to test these new parameters more conclusively from a statistical standpoint.

## Figures and Tables

**TABLE I:** Summary of New Quantitative Vessel Morphological Parameters for Differentiating Between Benign and Malignant Groups

Parameters	Benign (n = 35)	Malignant (n = 25)	P-value	Sen (%)	Sp (%)	AUC (%)	CI (%)
**SVP**	30/35	21/25	**1.04E-07**	84	86	85	[76 - 94]
**VDR**	0.70 ± 0.38	1.95 ± 0.62	**2.50E-06**	84	91	86	[74 - 98]
**mvFD**	1.13 ± 0.09	1.28 ± 0.03	**1.79E-05**	80	**92**	83	[72 - 96]
**MD Mean**	0.29 ± 0.13	0.40 ± 0.12	**2.39E-04**	**96**	78	**87**	[75 - 98]
**MD Median**	0.28 ± 0.15	0.41 ± 0.18	**2.22E-03**	77	69	73	[60 - 87]
**MD Max**	0.42 ± 0.19	0.68 ± 0.20	**1.37E-05**	80	78	83	[72 –94]
**MD Min**	0.19 ± 0.16	0.13 ± 0.16	3.58E-02	72	69	66	[51 - 81]
**BA Mean**	110.28 ± 24.21	95.86 ± 10.37	**5.28E-03**	91	76	85	[74 - 99
**BA Median**	108.70 ± 20.33	94.28 ± 12.38	1.39E-02	86	76	80	[68 - 97]
**BA Max**	127.36 ± 25.90	128.43 ± 22.99	7.93E-01	60	49	52	[34 - 74]
**BA Min**	95.84 ± 23.42	65.99 ± 18.13	**2.00E-04**	88	81	78	[74 - 99]

Sen = sensitivity, Sp = specificity, AUC = area under Curve and CI = confidence interval. The numbers for Sen, Sp, AUC and CI are given in percentile.
